# Flexural Behavior of BFRP Bar–Recycled Tire Steel Fiber-Reinforced Concrete Beams

**DOI:** 10.3390/ma17246197

**Published:** 2024-12-18

**Authors:** Jing-Hua Fu, Ao Zhang, Kai-Feng Chen, Bao-Yuan Li, Wei Wu

**Affiliations:** 1 National Engineering Research Center of Fiber Optic Sensing Technology and Networks, Wuhan University of Technology, Wuhan 430070, China; foryoufirst@whut.edu.cn; 2 China Construction Yipin Investment Development Co., Ltd., Wuhan 430000, China; 3 School of Civil Engineering and Architecture, Wuhan University of Technology, Wuhan 430070, China; ckfv50@whut.edu.cn; 4 Hubei Industrial Construction Group Co., Ltd., Wuhan 430065, China; hbgjz@126.com; 5 Urban Construction College, Wuchang Shouyi University, Wuhan 430064, China; 15271875775@163.com

**Keywords:** BFRP bars, fiber reinforcing, recycling tire steel fiber, bending performance, theoretical predictions

## Abstract

This research advocates for the use of basalt fiber-reinforced polymer (BFRP) bars and recycled tire steel fibers to reinforce concrete beams. Six concrete beams were constructed using different volume contents of recycled tire steel fibers (0, 0.5%, 1.0%, 1.5%) and BFRP reinforcement ratios (0.48%, 0.75%, 1.08%). Mechanical properties tests were conducted to investigate the flexural characteristics and failure modes of beams utilizing the non-contact full-field strain displacement measuring technology–digital image (3D-DIC) technology. The recycled tire steel fiber (RTSF) improves flexural performance, which contributes to inhibiting crack propagation, reducing flexural deformation, and improving the first cracking and ultimate loading capacities. Under the same reinforcement ratio, in comparison to ordinary BFRP beams, the cracking load of BFRP-RTSF beams increased by 17.73%, 23.76%, and 42.94%, respectively, and the ultimate bearing capacity increased by 4.03%, 5.85%, and 13.21%, respectively. In addition, a modified calculation model of bearing capacity considering RTSF tensile strength is proposed. The predicted values of BFRP-RTSF beams match well with the experimental values.

## 1. Introduction

The usage of BFRP for structural reinforcement has grown dramatically in recent years because of its excellent corrosion resistance, high temperature resistance, and freeze–thaw performance at a comparatively low cost [1]. The use of BFRP in building structures rather than traditional steel bars can effectively resolve the issue of steel corrosion. BFRP bars have the ability to considerably extend the lifespan of concrete structures and are prevalent in construction projects relating to marine environments.

However, during usage, FRP-reinforced concrete members typically exhibit greater deformation and crack breadth because of the smaller elastic modulus of FRP bars than steel bars [2,3,4,5,6]. According to relevant studies, incorporating steel fiber, basalt fiber, and polyolefin synthetic fiber into FRP bar-reinforced concrete will successfully postpone the formation of beam fractures, minimize the width of beam fractures, and increase beam ultimate flexural capacity and ductility [7,8]. By incorporating fiber into GFRP-reinforced lightweight aggregate concrete, concrete beams’ ultimate bearing capacity can be increased by 55% to 233% [9]. The addition of 0.5% steel fiber to the volume fraction can significantly enhance the ductility and flexural capacity of GFRP-reinforced concrete beams [10]. At present, more attention has been paid to the components formed by FRP bars and reinforced fibers, and the selected reinforced fibers are mainly industrial steel fibers, synthetic fibers, glass fibers, etc., without taking into account the usage of recycled tire steel fibers (RTSFs).

The pertinent literature for the RTSF investigation demonstrates that the mechanical characteristics of industrial steel fiber-reinforced concrete and RTSF-reinforced concrete are comparable [11,12,13]. Compared to concrete with industrial steel fibers (ISFs), RTSF concrete has superior tensile strength and ductility because of the various steel types utilized and the initial geometrical deformation of RTSFs [14]. The shape of RTSFs is more irregular than ISFs, which has a beneficial effect on its adhesion to concrete [12]. However, due to the irregular geometry of RTSFs, they show a stronger tendency to aggregate than other fibers; the amount of RTSFs needs to be controlled [15,16]. Adding RTSFs can avoid the brittle behavior of concrete [17,18]. At the same time, it is found that RTSFs can be used to completely replace ISFs [19,20]. Its production cost is only 5% of industrial steel fiber-reinforced concrete, which has a high economic benefit and environmental protection value [21]. The incorporation of RTSFs into regular concrete will enhance the material’s ductility, flexural toughness, and compressive strength [22,23]. However, an excessive, very short, or longer RTSF will have a negative impact on concrete [24,25]. Considering comprehensively, it is more ideal to use RTSFs as the fiber source of fiber-reinforced concrete [26]. When using RTSFs in beam components, RTSFs can enhance the bending resistance of beam components and improve their cracking performance after bending [7,9,27]. As BFRP bars possess traits of alkali resistance and cost-effectiveness, enhancing the mechanical properties of BFRP-reinforced concrete structures by substituting traditional fibers with RTSFs and evaluating its suitability holds paramount importance. This approach not only uplifts waste recycling, safeguarding the environment and promoting equitable development, but also resolves the issues of extensive cracks and frail failure of BFRP-reinforced concrete members when loaded.

Investigating the flexural properties of concrete beams reinforced with BFRP bars and recycled tire steel fibers is the main goal of this study. Six beam specimens with different RTSF volume contents (0%, 0.5%, 1.0%, 1.5%), as well as BFRP bar reinforcement ratios (0.48%, 0.75%, 1.08%), were assembled and evaluated. Using the 3D-DIC technology, a detailed analysis of the flexural performance, failure mode, crack distribution, and propagation process of BFRP-RTSF beams was carried out. The paper discusses the failure and crack development mechanism of BFRP-RTSF beams. The modified model of BFRP-RTSF beams is used to calculate the theoretical prediction value of the flexural performance of the test beam, which is then compared with the beam’s test data.

## 2. Materials and Methods

### 2.1. Recycled Tire Steel Fiber Concrete

#### 2.1.1. Raw Materials

**Figure 1:**
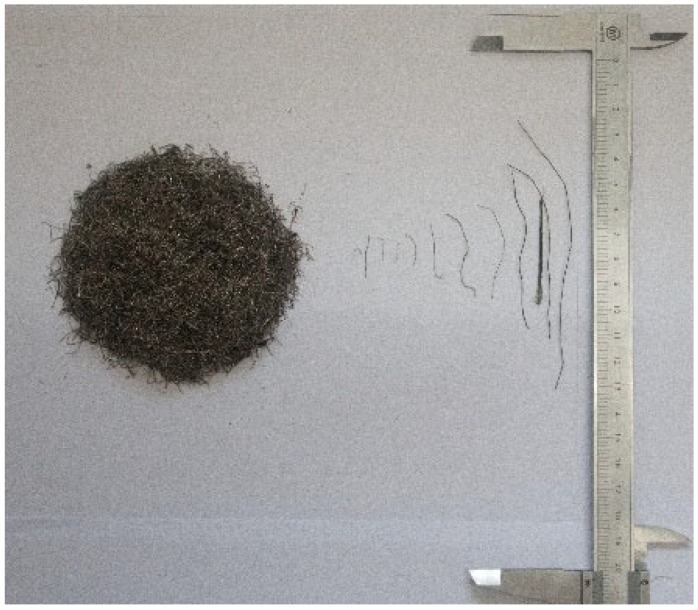
RTSF used in this study.

**Figure 2:**
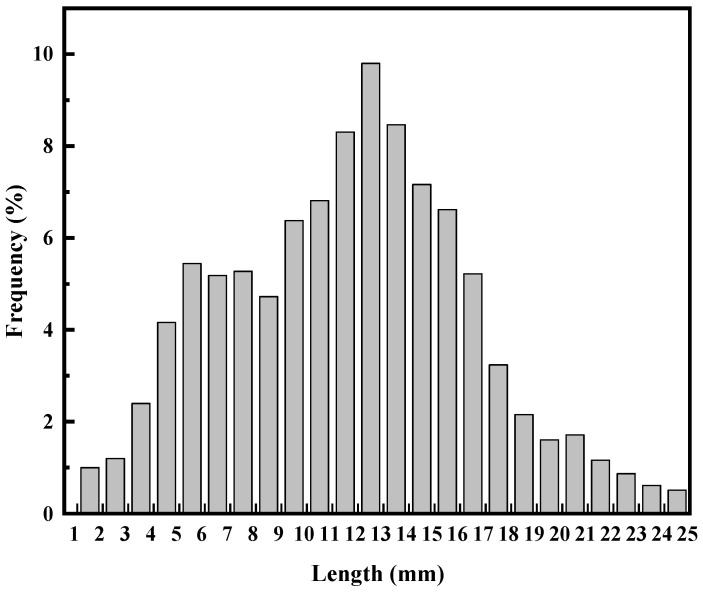
Length distribution of recycled tire steel fiber (RTSF).

The concrete mixture used in the experiment consisted of cement, coarse aggregate, a water-reducing agent, and fine aggregate. The cement used was P. O 42.5 ordinary Portland cement. The main chemical composition of the cement is shown in Table 1. Well-graded gravel with particle sizes ranging from 5 to 20 mm and less than 1% muck component formed the coarse aggregate. The fine aggregate was typical river sand with a fineness modulus of 2.80. The water-reducing agent was a polycarboxylate superplasticizer. The steel fiber is shown in Figure 1, obtained from disassembling waste tires, and it measures between 4 and 25 mm in length, 0.22 mm in diameter, and the length distribution is shown in Figure 2 and has an elastic modulus of 200 GPa and tensile strength of 2165 MPa.

#### 2.1.2. Mix Proportions

Table 2 shows the mixing ratio of raw materials in BFRP bar recycled tire steel fiber concrete. The RTSC represents recycled tire steel fiber-reinforced concrete, and the number represents RTSF volume content, for example (0.5%). After the dry material was mixed evenly, the recycled tire steel fiber was evenly spread into the mixer and continued with dry mixing for three minutes. After the steel fiber of the recycled tire was evenly distributed, the water and water reducer were added into the mixer for another three minutes of mixing. After the concrete mixing was completed, the concrete was poured into the mold and inserted into the vibrating rod for vibration. In order to prevent the steel fiber of the recycled tire from agglomeration, the vibration time should be reasonably controlled. The samples were then demolded after one day of curing, and the surface of the specimen was covered by felt and misted with water for curing until 28 days.

#### 2.1.3. Mechanical Performance

The fundamental mechanical characteristics of the designed concretes are illustrated in Table 3.

### 2.2. BFRP Bar

**Figure 3:**
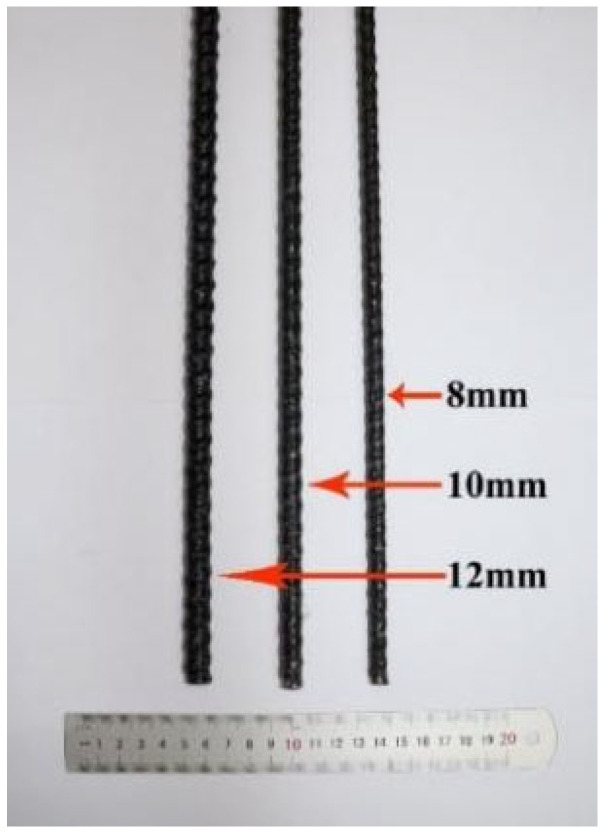
BFRP bar used in this study.

Figure 3 is a BFRP bar used for BFRP bar recycling tire steel fiber-reinforced concrete beams. BFRP bars are deep-threaded basalt bars. They are produced by Jiangsu Greenwood Valley New Material Technology Development Co., Ltd. (Nanjing, China). According to the information provided by the manufacturer, the length of BFRP bars is 1980 mm, the diameter is 8 mm, 10 mm, and 12 mm, respectively, the tensile strength (SD) is 1394 MPa (1.18%), 1403.4 MPa (1.21%), and 1230 MPa (1.17%), respectively, and the elastic modulus (SD) is 58.2 GPa (0.25%), 57.4 GPa (0.23%), and 46.2 GPa (0.26%), respectively.

### 2.3. Test Specimens

**Figure 4:**
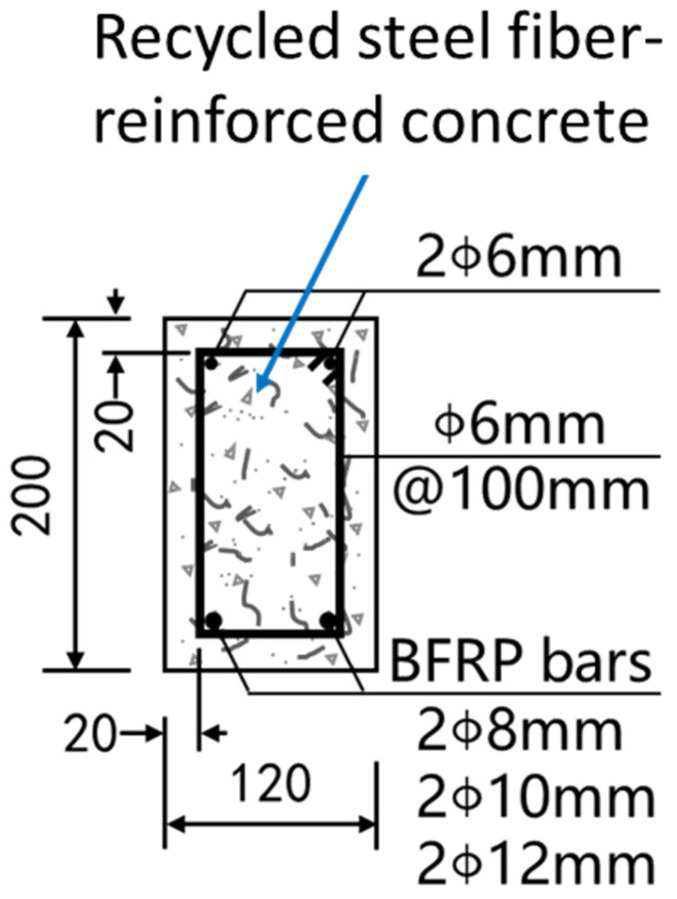
Details for the specimens.

In order to investigate the flexural performance of BFRP-RTSF beams, we designed and constructed five beams. Additionally, we fabricated one concrete beam without RTSF. Each beam is 120 mm by 200 mm by 2000 mm for its standard section, 20 mm for the thickness of the concrete cover, and 1800 mm for the calculated span. All specimens were constructed in a symmetrical manner, incorporating longitudinal steel bars. For the erecting reinforcement and stirrups, HPB300 steel bars with a diameter of 6 mm were employed. The erecting reinforcement refers to the longitudinal structural steel bar erected by the auxiliary stirrup. Its main function is to fix the stressed steel bar in the correct position and connect it with the stressed steel bar to form a steel bar skeleton so as to give full play to their respective mechanical properties. With a 6 mm diameter, HPB300 rebar has a tensile strength of 465 MPa and an elastic modulus of 202 GPa. The stirrup spacing was 100 mm, and in the pure bending section, stirrups were not used. Supplementary details and the arrangement of the beam are provided in Figure 4. The BRTSC represents BFRP-RTSF beams. This study focuses on the research parameters of the volume content of recycled tire steel fiber and the reinforcement ratio of BFRP bars. Among them, the RTSF volume content comprises four distinct values, which are 0%, 0.5%, 1.0%, and 1.5%. The diameter of BFRP bars is available in three sizes, 8 mm, 10 mm, and 12 mm. Table 4 displays the key parameters of the test beam design.

### 2.4. Testing Methods

#### 2.4.1. Four-Point Bending Loading Test

In accordance with the specifications set forth in GB/T50152-2012 [28], a 500 kN press was selected for the purpose of conducting a test on BFRP bar–recycled tire steel fiber-reinforced concrete beams. The loading was applied using the four-point bending loading method. The loading method employed was graded loading. The incremental loading was 5 kN. Upon reaching the calculated value of the cracking load, the loading step distance was altered to the 0.5 kN level, and the load holding period of each level was extended to five minutes.

**Figure 5:**
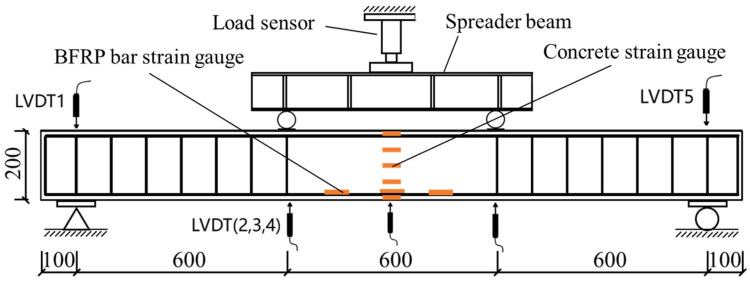
Four-point bending configuration for BFRP-RTSF beams (unit: mm).

In the course of the experiment, five linear variable differential transducers (LVDTs) and 11 strain gauges (six longitudinal gauges for steel and five for concrete) were employed for each specimen. Figure 5 illustrates the loading diagram and the configuration of the strain gauges and LVDTs. The load was applied by means of a 500 kN press. The entire process was conducted using displacement control. The configuration of the LVDTs and strain gauges, together with the loading diagram, are illustrated in Figure 5. The load was applied by a 500 kN press. The entirety of the experimental procedure, including the measurement of the data, was conducted under displacement control. The load was measured by a 50 t pressure sensor, and the resulting data were collected by a TST3826F-L static acquisition instrument. The data pertaining to displacement, strain, deflection, and crack formation were collected utilizing both a traditional measurement methodology and a three-dimensional digital image correlation (3D-DIC) approach.

#### 2.4.2. 3D-DIC Test

**Figure 6:**
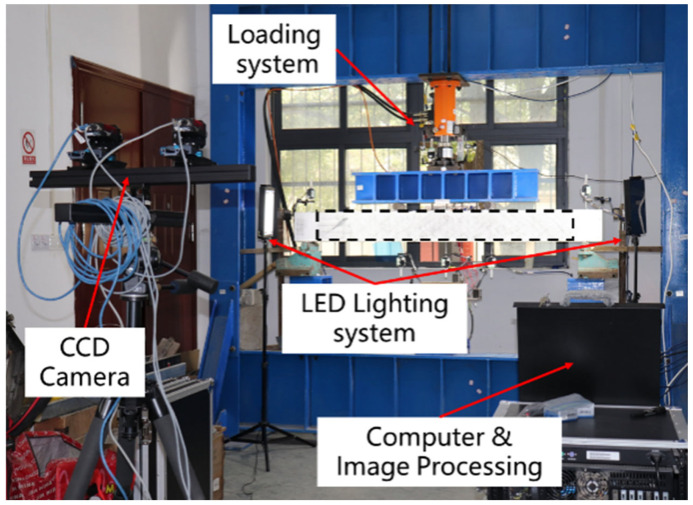
3D digital image correlation measurement system.

Scholars have already studied the combination of visualization technology and experimental methods [29], so in this paper, 3D-DIC visualization technology is used to test the cracking and bending performance of concrete beams. The data acquisition process makes use of 3D-DIC technology. Figure 6 depicts the configuration of the test system. The following section outlines the primary test steps: (1) speckle patterns were created on the surface of the test specimens with the objective of identifying and localizing the pixels of the collected images. (2) The test specimen was fixed in position, the tripod was adjusted to the requisite location, and the charge-coupled device (CCD) camera was erected. The brightness of the light source was adjusted, as was the aperture and focal length of the camera. The camera was then positioned to enable complete identification of the measurement area. Finally, the test equipment was fixed in place. (3) Utilizing the calibration board, the internal and external parameters of the binocular camera were obtained through calibration of the two CCD cameras. (4) Subsequent to loading, the CCD camera shutter was operated by VIC-Snap image acquisition control software (v 6.0) to capture an image of the test beam measurement area. (5) Subsequent to the completion of the test, the VIC-3D software (v 9.0) should be opened, the pertinent parameters set, the collected images calculated and analyzed, and the corresponding test results obtained.

## 3. Experimental Results and Discussion

### 3.1. Failure Mode and Crack Behavior

#### 3.1.1. Failure Mode

**Figure 7:**
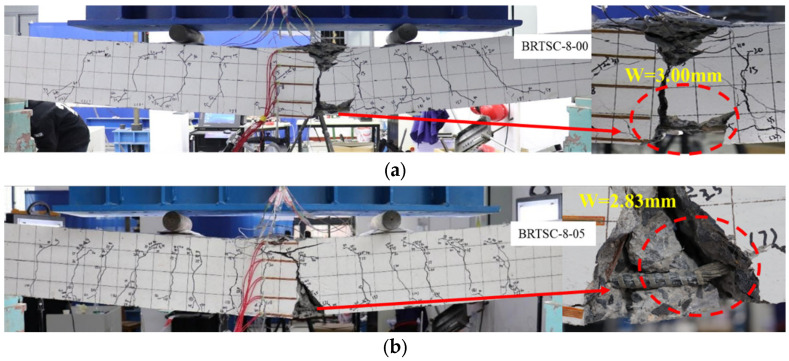

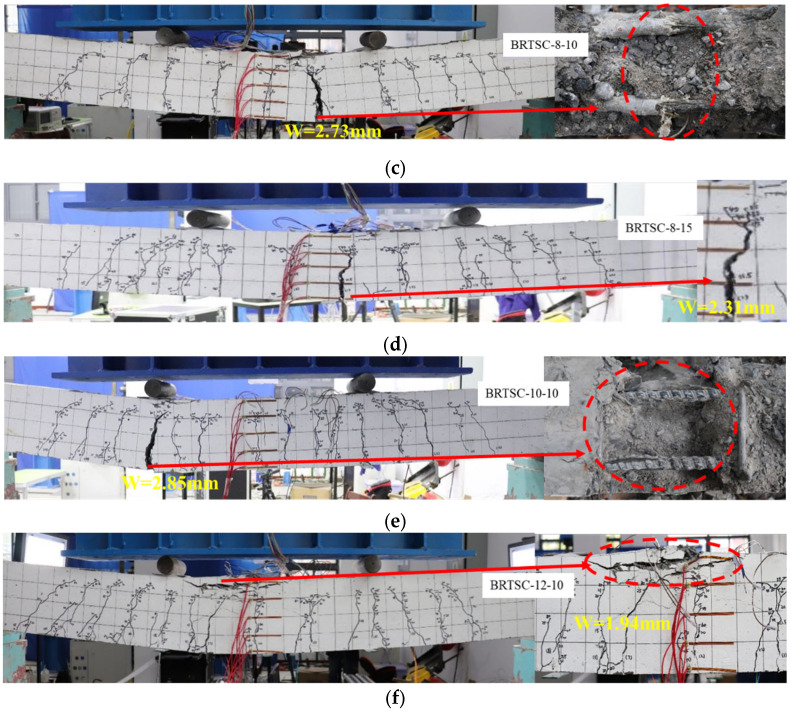
Crack patterns and failure of beams of BRTSC: (**a**) BRTSC-8-00; (**b**) BRTSC-8-05; (**c**) BRTSC-8-10; (**d**) BRTSC-8-15; (**e**) BRTSC-10-10; (**f**) BRTSC-12-10 (w represents crack width).

Figure 7 depicts the crack morphology and failure mode of BFRP-RTSF beams. Three failure modes have been identified, namely concrete crushing failure, equilibrium failure, and basalt reinforcement tensile rupture failure. The results show that the failure mode of BFRP-RTSF beams would be affected by the content of RTSF. BRTSC-8-00 is characterized by concrete crushing damage; when the content of RTSF increases, the failure mode of BRTSC-8-05 is equilibrium failure. The test beams with higher fiber content (BRTSC-8-10, BRTSC-8-15) showed compressive failure of concrete. When RTSF is added to the entire section of the specimen, the incorporation of RTSF enhanced the compression zone performance of the concrete [30]. It is evident from specimen BRTSC-10-10 that the failure position of the test beam may appear on both sides of the pure bending region, near the loading point. Other researchers have observed similar shear compression failure [7,31,32]. This is due to the depth expansion of cracks in FRP bar-reinforced beams.

Table 5 displays the results for the cracking load, collapse load, and failure mechanism of the test beam. The findings demonstrate an increase in the cracking load of the test beam with a rise in the volume content of RTSF. Compared with the control group BRTSC-8-00, the cracking loads of BRTSC-8-05, BRTSC-8-10, and BRTSC-8-15 increased by 22.36%, 22.97%, and 37.22%, respectively. This is due to the slightly curved shape of RTSF, which can better withstand tensile stress in the bending zone. Also, as the amount of RTSF content increases, the beam’s bearing performance can be enhanced, which is the same as previous research results [24]. The research of Htet et al. [33] shows that the ultimate load of concrete beams prepared with 0.67% recycled PF and 0.67% BF mixed fibers can be increased by 8.2% compared with that without fibers. In this study, the ultimate load of concrete beams can be increased by about 13% by adding RTSF. Therefore, compared with the mixed recycled PF and BF, the single addition of RTSF is more conducive to the improvement of the bending resistance of BFRP-reinforced concrete beams. The test beams’ cracking load was barely affected by changing the reinforcement ratio of BFRP bars. This is because the elastic modulus of FRP bars and concrete are comparable. These results indicate that enhancing the BFRP bars’ reinforcement ratio will increase the collapse load capacity of concrete beams. Compared with the control group BRTSC-8-10, the collapse bearing capacity of BRTSC-10-10 and BRTSC-12-10 increased by 29.61% and 46.99%, respectively.

This study indicates that compared to simply increasing the reinforcement ratio of BFRP bars, incorporating RTSF can considerably enhance the tensile strength of concrete in the tensile region. When the content of RTSF exceeds 0.5%, the failure mode changes from concrete crushing to BFRP bar cracking. That is because the incorporation of RTSF improves the performance of concrete in compression regions. Raising the reinforcement ratio can improve their flexural capacity of BFRP-RTSF beams.

#### 3.1.2. Cracking Behavior

**Figure 8:**
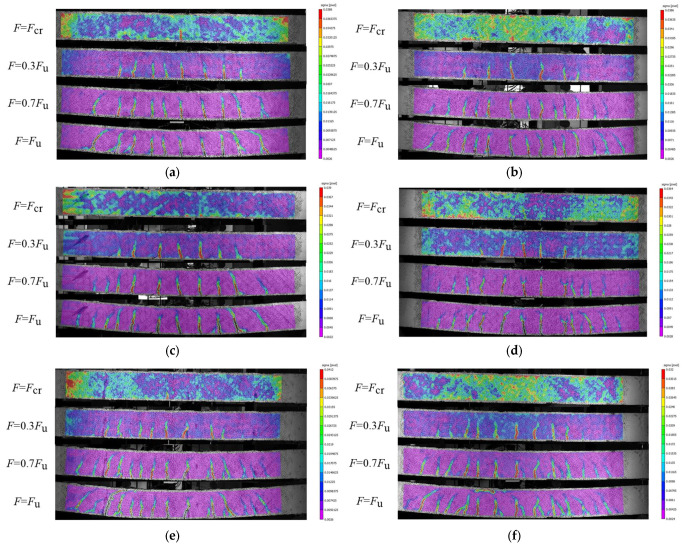
Crack development process of beams measured by 3D-DIC: (**a**) BRTSC-8-00; (**b**) BRTSC-8-05; (**c**) BRTSC-8-10; (**d**) BRTSC-8-15; (**e**) BRTSC-10-10; (**f**) BRTSC-12-10.

**Figure 9:**
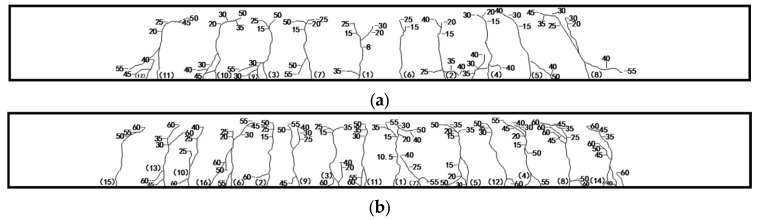

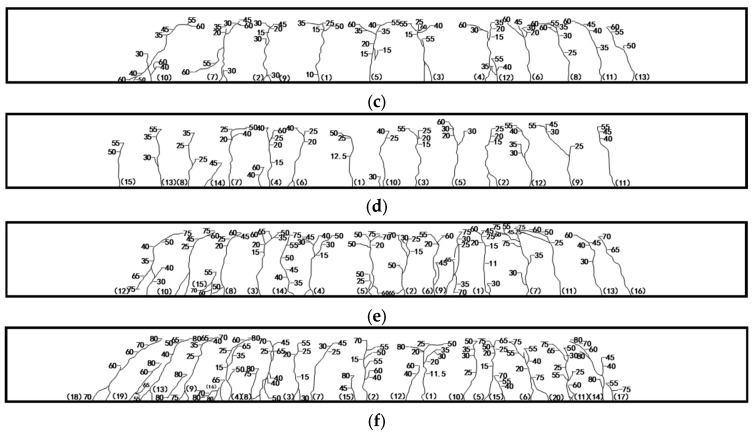
Crack development in beam under flexural loading: (**a**) BRTSC-8-00; (**b**) BRTSC-8-05; (**c**) BRTSC-8-10; (**d**) BRTSC-8-15; (**e**) BRTSC-10-10; (**f**) BRTSC-12-10. (The numbers in the figure represent the load values and the numbers in parentheses represent the order in which the cracks appeared).

Figure 8 shows how the 3D-DIC approach is utilized to record the surface crack development of each test beam under $F_{cr}$, 0.3 $F_{u}$, 0.7 $F_{u}$, and $F_{u}$ conditions during the bending process. Figure 9 details the process of crack development in BFRP-RTSF beams during loading. The figure’s numeral corresponds to the load value whereby crack propagation occurs. Figure 7, Figure 8 and Figure 9 demonstrate that the test beams’ surface crack development process typically comprises three stages. Upon reaching the cracking load, the first crack emerges in the pure bending region of the test beam, marking the commencement of the first stage. The number of cracks increases rapidly and extends upward to a higher position as the load increases. In the second stage, the rate of crack development slows as the load increases, and the number of cracks in the pure bending section stabilizes. Furthermore, an increasing number of cracks extend from the mid-span to the shear span section, displaying oblique characteristics. In the third stage, as the load increases beyond a certain point, the quantity of surface cracks on the beam remains constant. However, the width and height of the cracks continue to increase and subsequently extend into the beam’s compression region in the vicinity of the load application point. The rate of crack propagation accelerates when the load applied to the beam reaches its ultimate bearing capacity, which is caused by the upward movement of the neutral axis. This results in a reduction in the compression zone for the concrete, which subsequently undergoes crushing and ultimately leads to compression failure of the beam.

Under the same ratio of reinforcement with BFRP bars, the concrete beams with RTSF in the test group exhibit a greater rate of surface crack development. At 0.3 times the collapse load, the number of cracks approaches the total number of cracks in the limit state, while the height of the cracks is higher. As the volume content of RTSF increases, both the total number of cracks and the height decrease. When the RTSF content is 1.5%, its impact is most notable. The inclusion of recycled tire steel fiber in limiting crack development is primarily evident during the initial loading phase. This is because upon small loads, the RTSF disperses the tensile force on both sides of the micro-crack, playing an effective bridging role. During the process of tensile testing, the extraction of RTSF from the concrete matrix absorbs some energy and reduces stress concentration at the top of the crack. Under high loads and increasing crack widths, the RTSF begins to fracture or extract, with its resistance to cracking decreasing accordingly. As the ratio of basalt reinforcement increases, the speed and ultimate height of the crack extension are significantly reduced, effectively limiting the crack development process. At the same time, the crack spacing of the test beam with a high reinforcement ratio is smaller and the number of cracks is greater, with a more uniform distribution of cracks on the beam surface. That is because a high reinforcement ratio can increase the ultimate tensile strain of concrete, which leads to the improvement of crack resistance. Conversely, a higher reinforcement ratio can significantly decrease concrete strain concentration at the crack and better control the crack width. That is because the crack width during structural cracking is directly proportional to tensile strain. When cracking occurs, stress concentration is formed near the cracked zone. The higher the stress concentration, the larger the crack width under similar conditions. Within a specific range, increasing the steel fiber content and basalt reinforcement ratio enhances the crack resistance of concrete beams.

### 3.2. Load–Maximum Crack Width Response

**Figure 10:**
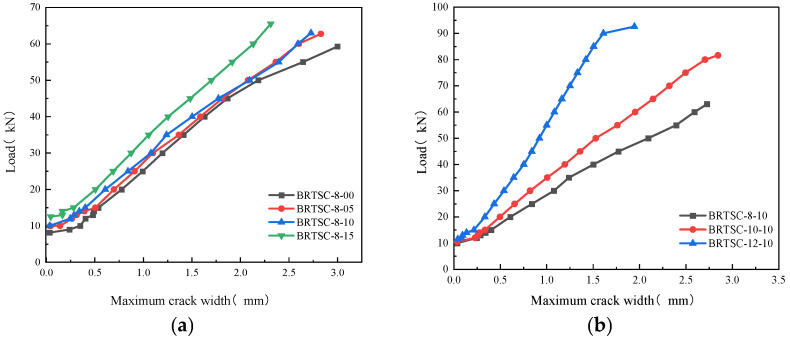
Load versus maximum crack width: (**a**) beams with different RTSF volume fractions; (**b**) beams with different BFRP reinforcement ratios.

Figure 10 depicts the association between load and the maximum crack width for the test beam. At first the specimen is not cracked, so the curve is a blank section. As the load increases to reach the cracking load, the concrete at the edge of the tensile region begins to crack. Despite exceeding the value of the cracking load, adhesion between the concrete and the reinforcement between the cracks exists, which is referred to as the tension stiffening phenomenon. After the load reaches about 15 kN, the concrete in the tensile region no longer works, causing the crack width to increase. In this stage, the initial crack grows as the load increases until the specimen is destroyed.

As indicated in Figure 10a, the addition of RTSF postpones the cracking of the test beam. The maximum crack width of the test beam rapidly reduces under the same strain as the volume fraction of RTSF increases. Once the volume content of RTSF reaches 1.5%, the growth rate of the maximum crack width of the test beam grows significantly slower than that of the BFRP-reinforced ordinary concrete beam. During the loading process, the tensile stress on the BFRP bar at the crack section decreases as the stress is shared by the BFRP bar on either side of the crack connected by the RTSF. This reduces the strain on the BFPR bar at the crack interface and effectively restricts the opening of the crack. Taking a load of 50 kN as an example, compared with BRTSC-8-00, the maximum crack width of BRTSC-8-05, BRTSC-8-10, and BRTSC-8-15 decreased by 4.77%, 4.05%, and 22.07%, respectively.

It is evident from Figure 10b that given the same load, the tensile strain of the high reinforcement ratio test beam is small and exhibits smaller crack width. For instance, at a load of 50 kN, the maximum crack widths of BRTSC-10-10 and BRTSC-12-10 are 27.02% and 56.03% lower than that of BRTSC-8-10, respectively. The maximum crack width of BRTSC-12-10 increases significantly with the approach to the ultimate load. This is because the presence of RTSF causes plastic deformation of the concrete in the compression region of the specimen near the ultimate load, which is gradually crushed. As a result, the maximum crack width is significantly increased. Thus, the impact of RTSF content is primarily reflected in the cracking load of concrete beams. The concrete in the area where tensile stress occurs gradually weakens over time, and the maximum width of cracks in concrete beams is primarily influenced by the amount of reinforcement. All these indicate that increasing the amount of RTSF and raising the reinforcement ratio can delay the growth of the crack width of concrete beams.

### 3.3. Load–Deflection Response at Mid-Span

**Figure 11:**
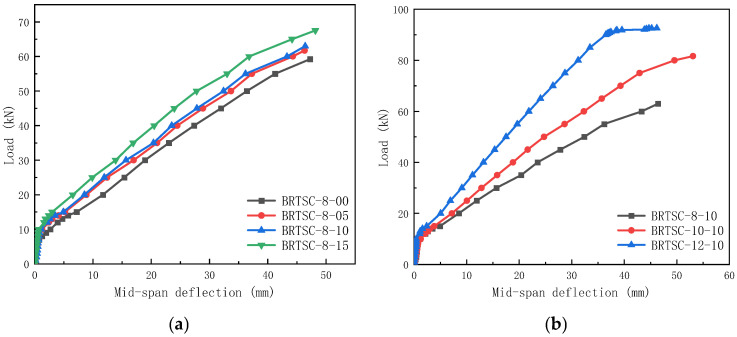
Load–deflection response at mid-span for concrete beams: (**a**) beams with different RTSF volume fractions; (**b**) beams with different BFRP reinforcement ratios.

The load–mid-span deflection curve for BFRP-RTSF beams is shown in Figure 11. The BFRP-RTSF beam’s load–mid-span deflection curve exhibits bilinear deformations, as shown in Figure 11. Figure 11a illustrates that under the same reinforcement ratio, each test beam initially withstands a combined action of concrete and BFRP bars to resist deformation, resulting in only small deformations. At this stage, the load–mid-span deflection curve exhibits a steep slope, indicating a significant stiffness of the specimen. Once the load surpasses the cracking load, the initial crack appears in the middle of the beam span. Consequently, the bending stiffness diminishes as the concrete in the region of the maximum bending moment reaches its tensile threshold and ceases to contribute to the load-bearing capacity. As a result, the tensile BFRP bars take on the whole load-bearing duty and the load curve’s slope begins to decrease. With increasing load, the specimens demonstrate a notable increase in the number and size of cracks with deformation. There is no obvious yield point for the specimens because of the near-linear elastic stress and strain of BFRP bars without a noticeable yield extent. As a result, the BFRP bars yield and the concrete in the compression zone of the beam breaks and immediately collapses. Therefore, the mid-span deformation exhibits consistent growth up until the limit deformation is reached. As a result of the crack resistance offered by the RTSF, the stiffness of BFRP bar–RTSF-reinforced concrete beams are enhanced upon cracking compared to that of BFRP-reinforced ordinary concrete beams. The mid-span ultimate deflection of BRTSC-8-05, BRTSC-8-10, and BRTSC-8-15 increased by 3.23%, 3.48%, and 7.33%, respectively. RTSF considerably restricts the development of the mid-span deflection of BFRP bar–RTSF-reinforced concrete beams when the load exceeds 20 kN. The load–mid-span deflection curve slope of the test beam gradually increases as the volume content of RTSF increases. Meanwhile, the mid-span deflection of the beam decreases under the same load as the volume content of the RTSF increases. All these indicate that RTSF can limit the deflection development of BFRP-RTSF beams, and it is advantageous to fully use the bending resistance of FRP beams.

It is evident from Figure 11b that the load–deflection deformation of the testing beam exhibits a comparable trend to that of the specimen with the same reinforcement ratio. Initially, when subjected to a low load, the testing beams resist deformation via the joint mechanism of concrete and BFRP bars, resulting in a sharp slope of the load–mid-span deflection curve. The concrete in the test beam cracks and withdraws from the work as the load approaches the breaking point, reducing the specimen’s stiffness until the longitudinal reinforcement yields. The ultimate deflection of BRTSC-10-10 increased by 14.54%, while the ultimate deflection of BRTSC-12-10 decreased by 0.5%. When the load is 20 kN, the mid-span deflection is reduced by 15.57% and 40.59% compared to the BFRP-RTSF beam with a reinforcement ratio of 0.48%. Among them, the BFRP-RTSF beam utilizing RTSF concrete with a high basalt reinforcement ratio of 1.08% exhibits a horizontal section in its load–mid-span deflection curve after the load exceeds 90 kN. This demonstrates that the RTSF concrete’s plastic deformation capacity is fully utilized in the compression region, with the test beam displaying certain ductility characteristics even upon failure. At the same time, it is notable that the BFRP bar’s reinforcement ratio has minimal impact on the load–mid-span deflection curve slope before the test beams’ cracking. Also, each test beam’s initial stiffness is comparable. When the load reaches the cracking point, the reinforcement ratio of the basalt reinforcement leads to an increase in the test beam’s stiffness. Comparing Figure 11a,b, it is evident that under the same load, increasing the BFRP reinforcement ratio is more effective in reducing mid-span deflection and bearing a greater load capacity instead of increasing the volume content of steel fiber. This is a result of the fact that concrete becomes inactive after cracking, with only a small portion of steel fiber providing bending resistance. By increasing the reinforcement ratio, the specimen’s bending stiffness is improved. The addition of fiber serves as extra reinforcement that restricts the spread of current cracks, ultimately hindering the loss of beam stiffness. This outcome is lessened as the reinforcement ratio rises, making the reinforcement ratio essentially the main factor controlling post-crack response. Consequently, the impact of steel fiber content is chiefly manifested in the initial stage of the concrete, serving as the primary load-bearing structure before any signs of cracking appear. The flexural performance of the beam is significantly impacted by the reinforcement ratio when cracks develop in the tension zone.

### 3.4. Load–Strain Response of Concrete

**Figure 12:**
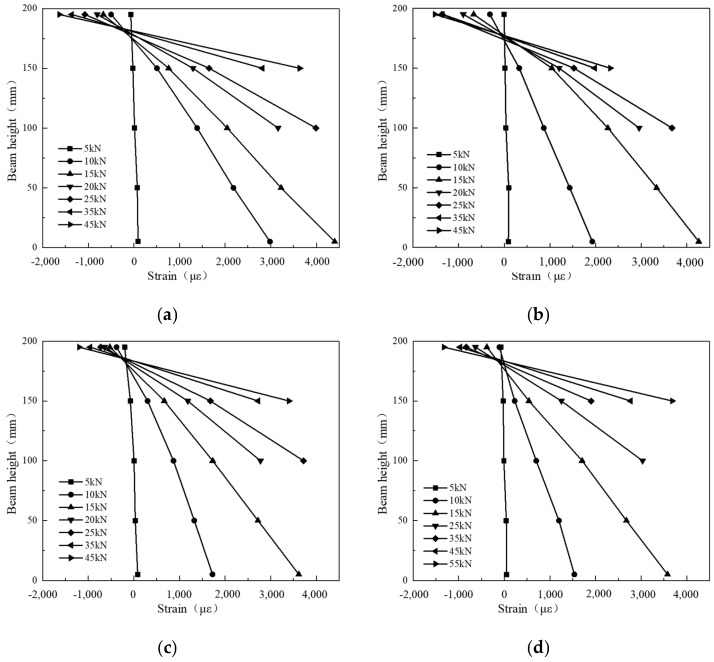

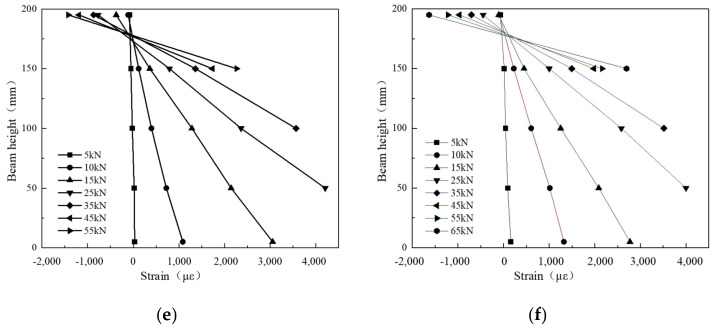
Strains of concrete at different positions along the height of beams under various loads: (**a**) BRTSC-8-00; (**b**) BRTSC-8-05; (**c**) BRTSC-8-10; (**d**) BRTSC-8-15; (**e**) BRTSC-10-10; (**f**) BRTSC-12-10.

The mid-span portion of the test beam was used to measure the concrete strains during bending using the 3D-DIC method in this experiment. The main purpose of the test was to study the section of the concrete beam that is always flat when it is bent, that is, to verify the assumption of the flat section, and to provide the basic assumption for the subsequent calculation of the theoretical bearing capacity of the concrete beam. This study refers to the existing literature [34]. In order to study the mid-span section of the beam, we arranged the strain gauges evenly along the height of the beam. Considering that the specimens on both sides of the upper and lower edges of the beam were not smooth, the strain gauges were arranged at a distance of 2mm from the edge of the beam. Measurements were taken at 2 mm, 50 mm, 100 mm, 150 mm, and 198 mm from the bottom of the mid-span of the beams. Concrete strains of the six test beams under different loads are depicted in Figure 12. Figure 12 indicates that the trend of concrete strain at various heights is comparable for all beams under different loads, where the upper part experiences compression and the lower part experiences tension. The neutral layer can be observed above the high center line of the beams. Initially, it was assumed that the strain from top to bottom along the beam height was almost linear, regardless of the loading level. This implies that the interaction between the concrete and BFRP bars is strong and that the beam cross-section remains flat during bending. This also suggests that concrete and BFRP bars can distribute the load effectively. Thus, this study can be utilized as a flat cross-section assumption for determining the flexural capacity of beams.

### 3.5. Load–Strain Response of BFRP Bar at Mid-Span

**Figure 13:**
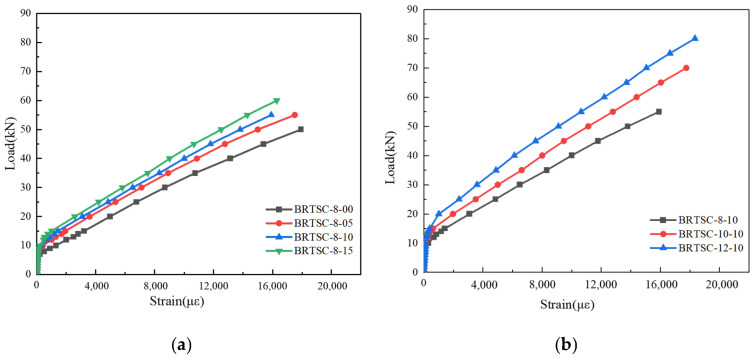
Load–strain response at the mid-span of basalt reinforcement in beams: (**a**) beams with different RTSF volume fractions; (**b**) beams with different BFRP reinforcement ratios.

The load-strain relationship of the BFRP bar at the mid-span of the tested beam is illustrated in Figure 13. Prior to the beam reaching its tensile strength and cracking, the load–strain curve at the mid-span demonstrates a rapid increase in the BFRP bar’s strain. This is due to the fact that during the initial phase of the test, the concrete and BFRP bar are pulled together, resulting in minimal strain for the BFRP bar, which remains nearly at zero. In the test beam, the tensile zone’s RTSF-reinforced concrete area is significantly larger than that of the BFRP bar. Furthermore, both materials exhibit a similar order of magnitude for their elastic modulus, thereby resulting in the BFRP bar undergoing a more rapid increase in strain compared to that of the RTSF-reinforced concrete. The stress on the concrete in the tensile zone increases in proportion to the applied load and ultimately reaches its limit. This causes the edge of the zone to either crack or the stress to redistribute. The BFRP bars then take on the tensile stress, resulting in a slow growth of the curve. After the crack, the cracks in the concrete expand and withdraw from the work.

It is evident from Figure 13a that under the same reinforcement ratio of BFRP bars, the inflection point of the load–strain of the BFRP bar at the mid-span relationship curve of the test beam is gradually delayed as the volume fraction of RTSF increases. This mainly occurs because incorporating RTSF increases the cracking load of the test beam, thus delaying the time when the BFRP bars bear the main tensile stress. After the test beam cracked, the stress provided by the RTSF increases with the increase in the volume fraction of RTSF, and the deformation of the BFRP bar decreases. Compared to BFRP-reinforced ordinary concrete beams, the strain of BFRP under the ultimate load of BFRP-RTSF beams with fiber content of 0.5%, 1.0%, and 1.5% decreases by 2.34%, 11.26%, and 9.15%, respectively. The strain of the BFRP bar decreases with the increase in fiber content under the same load.

Figure 13b illustrates that an increase in the reinforcement ratio of BFRP bars has a negligible impact on the initial phase of the load–strain behavior of BFRP bars at the mid-span curve of the test beam, when the volume content of RTSF remains constant. Nevertheless, following the formation of cracks in the test beam, the slope of the load–strain relationship curve for the BFRP bar at mid-span exhibits a notable increase with the reinforcement ratio of the BFRP bars. The BFRP bar experiences less deformation at the same load increment when the reinforcement ratio is high, leading to a significant increase in the curve’s slope. Relative to BFRP-RTSF beams with a reinforcement ratio of 0.48%, the BFRP strain increases by 28.65% and 32.89% for BFRP-RTSF beams with reinforcement ratios of 0.75% and 1.08%, respectively. Due to the increased reinforcement ratio, the bearing capacity of the specimen becomes greater, while the deformation also increases, indicating a strong bonding performance between BFRP bars and concrete before the final failure. Under the same load, as the reinforcement ratio increases and the tensile force of each BFRP bar decreases, resulting in smaller strain. As shown in Figure 13a,b, an increase in fiber content leads to an increase in the tensile stress provided by the fiber, causing a decrease in the tensile stress provided by the BFRP bar for the same reinforcement ratio. When the fiber content remains constant, the stress provided by BFRP decreases with the increase in the reinforcement ratio under the same load increment. Furthermore, concrete in the tensile zone withdraws from the work after the specimen cracks. The BFRP bars primarily bear the load, and the steel fiber only experiences a minimal amount of tensile stress. Therefore, increasing the steel fiber content and BFRP reinforcement ratio can effectively decrease the BFRP strain. However, a higher BFRP reinforcement ratio can more effectively improve flexural performance and increase the ultimate bearing capacity. The steel fiber content primarily delays the time when BFRP starts to bear tensile stress alone. When the concrete in the tensile region is fully ineffective, the BFRP stress is primarily influenced by the reinforcement ratio.

## 4. Comparison with Theoretical Previsions

The guidelines for steel-reinforced concrete beams may not apply to BFRP-RTSF beams, since the performance of FRP bars differs from conventional steel bars [35]. A new calculation model for the flexural capacity of BFRP-reinforced recycled tire steel fiber-reinforced concrete beams is necessary to be proposed. Reference to relevant studies [36], based on the following basic assumptions, a calculation method for the flexural capacity of basalt-reinforced RTSF-reinforced concrete beams under compression and tension failure is proposed [37,38]:

(1) The cross-section of the test beam remains flat during the whole failure process.

(2) The tensile stress of RTSF-reinforced concrete is considered. The tensile stress diagram of the RTSF of recycled tires in the tensile zone can be condensed as an analogous rectangular stress diagram.

(3) BFRP bars’ stress–strain relationship is linear. The following formula depicts the stress–strain relationship:

(1) $$\sigma_{f}=E_{f}\varepsilon_{f}$$

where $\sigma_{f}$, $E_{f}$, and $\varepsilon_{f}$ represent the tensile stress, elastic modulus, and tensile strain of basalt reinforcement, respectively.

(4) The compressive stress–strain relationship of RTSF-reinforced concrete is shown below [33].

(2) $$\sigma_{c}=\left\{\begin{array}{cc} f_{c}\left[1-{\left(1-\varepsilon_{c}/\varepsilon_{0}\right)}^{2}\right] & \varepsilon_{c}\leq \varepsilon_{0}\\ f_{c} & \varepsilon_{0}\leq \varepsilon_{c}\leq \varepsilon_{cu} \end{array}\right.$$

where $\sigma_{c}$, $f_{c}$, and $\varepsilon_{c}$ represent the compressive stress, axial compressive strength, and compressive strain of concrete, respectively; $\varepsilon_{0}$ is the compressive strain when the concrete reaches the axial compressive strength. For RTSF concrete, it is calculated according to Equation (3). $\varepsilon_{cu}$ is concrete’s ultimate compressive strain, which is 0.0045 for recycled tire steel fiber concrete.

(3) $$\varepsilon_{0}=0.0007V_{RTSF}\frac{l_{RTSF}}{d_{RTSF}}+0.0021$$

where $V_{RTSF}$ is the volume content of RTSF and $\frac{l_{RTSF}}{d_{RTSF}}$ is the aspect ratio of RTSF, which is 58.27.

(5) The bond–slip effect between BFRP bars and concrete and the influence of erecting bars in the compression zone are ignored.

### 4.1. Solution of Balanced Reinforcement Ratio

The concrete in the compression zone and the BFRP bars in the tension zone are destroyed simultaneously when the reinforcement ratio of BFRP bars exceeds a certain limit value, which is known as the equilibrium failure. The equilibrium conditions of the beam section force are

(4) $$b\frac{x}{\varepsilon_{cu}}\int_{0}^{\varepsilon_{cu}}\sigma_{c}(\varepsilon_{c})d\varepsilon_{c}=f_{fy}\rho_{b}h_{0}b+\sigma_{rtsf}(h-x)b$$

(5) $$\frac{x}{h_{0}}=\frac{\varepsilon_{cu}}{\varepsilon_{cu}+\varepsilon_{fy}}$$

where $\rho_{b}$ is the equilibrium reinforcement ratio, x is the height of the concrete compression zone, $f_{fy}$ is the nominal yield strength of the BFRP bar, taking $f_{fy}=0.8f_{fu}$, $\varepsilon_{cu}$ is the ultimate compressive strain of the concrete, $\varepsilon_{fy}$ is the strain when the BFRP bar reaches the nominal yield strength, taking $\varepsilon_{fy}=0.8\varepsilon_{fu}$, and $\sigma_{rtsf}$ is the equivalent tensile stress of the RTSF concrete in the tensile zone.

The simultaneous Equations (4) and (5) can be solved.

(6) $$\rho_{b}=\frac{\int_{0}^{\varepsilon_{cu}}\sigma_{c}(\varepsilon_{c})d\varepsilon_{c}}{f_{fy}(\varepsilon_{cu}+\varepsilon_{fy})}-\frac{\sigma_{rtsf}}{f_{fy}h_{0}}(h-\frac{\varepsilon_{cu}h_{0}}{\varepsilon_{cu}+\varepsilon_{fy}})$$

### 4.2. Calculation Model of Bearing Capacity of Compressive Failure

**Figure 14:**
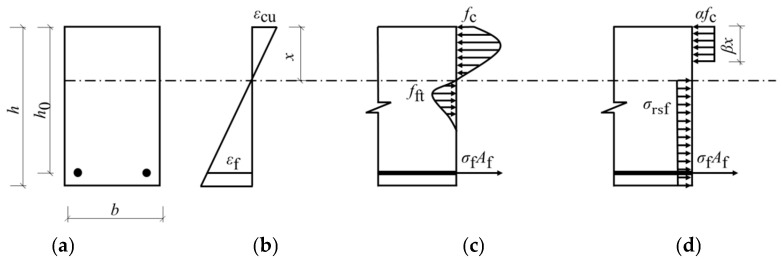
The calculation diagram of the flexural capacity of the beam section: (**a**) section; (**b**) strain distribution; (**c**) stress distribution; (**d**) equivalent rectangular stress distribution.

The beam shows compression failure if the actual reinforcement ratio exceeds $\rho_{b}$. When compression failure occurs, the calculation diagram of the flexural bearing capacity of the beam section is as shown in Figure 14.

From the cross-section equilibrium conditions, the following can be obtained:

(7) $$\alpha f_{c}\beta xb=\sigma_{f}A_{f}+\sigma_{rtsf}(h-x)b$$

(8) $$\frac{\varepsilon_{cu}}{\varepsilon_{f}}=\frac{x}{h_{0}-x}$$

where $\alpha f_{c}$ is the equivalent stress value of the equivalent rectangular stress diagram, where $\alpha =\frac{\int_{0}^{\varepsilon_{cu}}\sigma_{c}(\varepsilon_{c})d\varepsilon_{c}}{\beta \varepsilon_{cu}f_{c}}$; βx is the equivalent height, where $\beta =2(1-\frac{\int_{0}^{\varepsilon_{cu}}\sigma_{c}(\varepsilon_{c})\varepsilon_{c}d\varepsilon_{c}}{\varepsilon_{cu}\int_{0}^{\varepsilon_{cu}}\sigma_{c}(\varepsilon_{c})d\varepsilon_{c}})$; $A_{f}$ is the cross-sectional area of BFRP bars; h is the beam height; and $\sigma_{rtsf}$ is the equivalent tensile stress of RTSF concrete in the tensile zone and $\sigma_{rtsf}=\mu V_{RTSF}\frac{l_{RTSF}}{d_{RTSF}}$ is taken [37], where μ is the influence coefficient related to RTSF and 0.3882 is taken according to the test results.

The simultaneous Equations (7) and (8) can be solved.

(9) $$x=\frac{\sigma_{rtsf}hb-A_{f}E_{f}\varepsilon_{cu}+\sqrt{{\left(A_{f}E_{f}\varepsilon_{cu}-\sigma_{rtsf}hb\right)}^{2}+4A_{f}E_{f}\varepsilon_{cu}h_{0}\left(\alpha f_{c}\beta b+\sigma_{rtsf}b\right)}}{2\left(\alpha f_{c}\beta b+\sigma_{rtsf}b\right)}$$

The bearing capacity of the BFRP-RTSF beam under compression failure is

(10) $$M_{u}=\alpha f_{c}\beta xb\left(h_{0}-\frac{1}{2}\beta x\right)-\sigma_{rtsf}\left(h-x\right)\left(\frac{h-x}{2}-a\right)b$$

where a is the distance between the resultant force point of the BFRP bar and the concrete at the bottom edge.

### 4.3. Calculation Model of Bearing Capacity of Tensile Failure

If the actual reinforcement ratio is less than $\rho_{b}$, the beam is subjected to tensile failure. Similarly, according to the equilibrium conditions of the section force, the following is obtained:

(11) $$b\frac{x}{\varepsilon_{c}^{\prime }}\int_{0}^{\varepsilon_{c}^{\prime }}\sigma_{c}(\varepsilon_{c})d\varepsilon_{c}=f_{fy}A_{f}+\sigma_{rtsf}(h-x)b$$

(12) $$x=\frac{\varepsilon_{c}^{\prime }h_{0}}{\varepsilon_{c}^{\prime }+\varepsilon_{fy}}$$

where $\varepsilon^{\prime }$ is the compressive strain of the concrete at the top of the beam during tensile failure.

Combining Equations (11), (12) and (2), we obtain $\varepsilon^{\prime }$.

The bearing capacity of the BFRP-RTSF beam is

(13) $$M_{u}=\alpha f_{c}\beta xb\left(h_{0}-\frac{1}{2}\beta x\right)-\sigma_{rtsf}\left(h-x\right)\left(\frac{h-x}{2}-a\right)b$$

where $\alpha =\frac{\int_{0}^{\varepsilon_{c}^{\prime }}\sigma_{c}(\varepsilon_{c})d\varepsilon_{c}}{\beta \varepsilon_{c}^{\prime }f_{c}}$, $\beta =2(1-\frac{\int_{0}^{\varepsilon_{c}^{\prime }}\sigma_{c}(\varepsilon_{c})\varepsilon_{c}d\varepsilon_{c}}{\varepsilon_{c}^{\prime }\int_{0}^{\varepsilon_{c}^{\prime }}\sigma_{c}(\varepsilon_{c})d\varepsilon_{c}})$, and $x=\frac{\sigma_{rtsf}hb-A_{f}E_{f}\varepsilon_{c}^{\prime }+\sqrt{{\left(A_{f}E_{f}\varepsilon_{c}^{\prime }-\sigma_{rtsf}hb\right)}^{2}+4A_{f}E_{f}\varepsilon_{c}^{\prime }h_{0}\left(\alpha f_{c}\beta b+\sigma_{rtsf}b\right)}}{2\left(\alpha f_{c}\beta b+\sigma_{rtsf}b\right)}$.

The flexural capacity of six groups of test beams is calculated using the methods for estimating the flexural capacity of BFRP bar–recycled tire steel fiber-reinforced concrete beams under various failure modes suggested above. Table 6 displays the compared results of the theoretical calculation results and the test results.

From Table 6, it can be seen that the experimental and theoretical calculation values for the test beam’s flexural capacity coincide well, and the calculation stability is satisfactory. It shows that on the basis of considering the influence of RSTF, the proposed calculation method of the flexural capacity of BFRP bar–recycled tire steel fiber-reinforced concrete beams can better predict the flexural capacity of concrete beams under different failure modes.

## 5. Conclusions

In this paper, we proposed a novel type of BFRP bar–steel fiber-reinforced concrete member utilizing RTSF for enhanced environmental sustainability and cost-effectiveness. To evaluate its potential, we designed and fabricated concrete beams with varying steel fiber contents and reinforcement ratios. Subsequently, we conducted a flexural test on the beams and performed a crack analysis utilizing 3D-DIC technology. The study investigated how the mechanical properties and failure modes of concrete beams were affected by the RTSF content and reinforcement ratio of BFRP bars. Furthermore, a formula for calculating the bearing capacity of BFRP-RTSF beams was proposed. The main conclusions are as follows:

The study revealed that BFRP bar–recycled tire steel fiber-reinforced concrete beams exhibit flexural failure modes such as concrete crushing, equilibrium failure, and BFRP reinforcement tensile rupture failure. Increasing the volume of RTSF shows a bigger impact on enhancing the cracking load of the test beam than increasing the reinforcement ratio of BFRP bars. The test beam’s ultimate bearing capacity can be improved more significantly by increasing the BFRP bar’s reinforcement ratio.

Additionally, stiffness of the testing beam increases and the mid-span deflection decreases as the volume content of RTSF and the BFRP bar reinforcement ratio increase, all while under the same load after cracking has occurred. The mid-span deflection with a reinforcement ratio of 1.08% is only 50% of that with a reinforcement ratio of 0.48%. By boosting the volume content of the RTSF and BFRP reinforcement ratio, the average crack width and difference in crack width of the pure bending section of the beam decreases.

According to existing theory, the theoretical ultimate flexural capacity of BFRP bar–recycled tire steel fiber-reinforced concrete beams is similar to the measured results. The calculation model reliably predicts the flexural capacity of recycled tire steel fiber-reinforced concrete beams reinforced with BFRP bars. This finding is informative for practical engineering applications.

## Figures and Tables

**Table 1:** Main chemical composition of cement (%).

**Materials**	CaO	MgO	Al_2_O_3_	SiO_2_	Fe_2_O_3_	SO_3_	Na_2_O
**Cement**	60.56	2.22	5.98	21.35	2.91	2.05	0.21

**Table 2:** Mix proportion of raw materials in recycled tire steel fiber-reinforced concrete (kg/m^3^).

Concrete Mix	Water	Cement	Fine Aggregate	Coarse Aggregate	Superplasticizer	Fiber	Fiber Volume Fraction
RTSC-00	200	477	485	1283	4.0	0	0%
RTSC-05						39	0.5%
RTSC-10						78	1.0%
RTSC-15						117	1.5%

**Table 3:** Concrete properties.

NO.	Cube Compression Strength (MPa)/SD (%)	Prism Compressive Strength (MPa)/SD (%)	Split Tensile Strength (MPa)/SD (%)	Elastic Modulus (GPa)/SD (%)
RSFC-00	44.7/1.91	35.2/1.33	3.52/0.092	32.6/0.44
RSFC-05	49.5/1.9	39.1/1.29	3.86/0.09	34.5/0.39
RSFC-10	50.9/1.88	40.2/1.31	4.34/0.095	34.7/0.36
RSFC-15	51.4/1.86	40.7/1.3	4.83/0.09	34.9/0.41

**Table 4:** Specimen design details.

Specimen	Longitudinal Rib	Rebar Ratio/$\rho_{f}$ (%)	Volume Fraction/$V_{f}$ (%)
BRTSC-8-00	2Φ8	0.48	0
BRTSC-8-05	2Φ8	0.48	0.5
BRTSC-8-10	2Φ8	0.48	1.0
BRTSC-8-15	2Φ8	0.48	1.5
BRTSC-10-10	2Φ10	0.75	1.0
BRTSC-12-10	2Φ12	1.08	1.0

**Table 5:** Failure results of BRTS concrete beams.

Specimen	Cracking Load (kN)	Ultimate Load (kN)	Mid-Span Displacement (mm)	Failure Mode
BRTSC-8-00	8.14	59.24	46.26	Concrete crushing
BRTSC-8-05	9.96	62.74	43.31	Concrete crushing and FRP bar rupture
BRTSC-8-10	10.01	62.99	53.03	FRP bar rupture
BRTSC-8-15	11.17	67.52	48.16	FRP bar rupture
BRTSC-10-10	10.05	81.64	53.08	Concrete crushing
BRTSC-12-10	11.51	92.59	46.20	Concrete crushing

**Table 6:** Comparison of experimental values and calculated values of flexural capacity of test beam.

Specimen Number	Reinforcement Ratio (%)	Balanced Reinforcement Ratio (%)	Test Value (kN)	Calculated Value (kN)	T/C
BRTSC-8-00	0.48	0.37	58.77	53.48	1.10
BRTSC-8-05	0.48	0.54	61.14	62.15	0.98
BRTSC-8-10	0.48	0.54	62.21	63.42	0.98
BRTSC-8-15	0.48	0.53	66.53	64.20	1.04
BRTSC-10-10	0.75	0.53	80.16	78.73	1.02
BRTSC-12-10	1.08	0.61	87.99	82.65	1.06
Average value					1.03
Standard deviation					0.05

## Data Availability

All data, models, and code generated or used during the study appear in the published article.
